# Establishment of Landslide Groundwater Level Prediction Model Based on GA-SVM and Influencing Factor Analysis

**DOI:** 10.3390/s20030845

**Published:** 2020-02-05

**Authors:** Ying Cao, Kunlong Yin, Chao Zhou, Bayes Ahmed

**Affiliations:** 1 Faculty of Engineering, China University of Geosciences, Wuhan 430074, China; yingcao@cug.edu.cn; 2 School of Geography and Information Engineering, China University of Geosciences, Wuhan 430078, China; 3 Institute for Risk and Disaster Reduction, University College London (UCL), Gower Street, London WC1E 6BT, UK; bayesahmed@gmail.com

**Keywords:** landslides monitoring, groundwater level prediction, Support Vector Machine, influencing factors, Three Gorges Reservoir area

## Abstract

The monitoring and prediction of the landslide groundwater level is a crucial part of landslide early warning systems. In this study, Tangjiao landslide in the Three Gorges Reservoir area (TGRA) in China was taken as a case study. Three groundwater level monitoring sensors were installed in different locations of the landslide. The monitoring data indicated that the fluctuation of groundwater level is significantly consistent with rainfall and reservoir level in time, but there is a lag. In addition, there is a spatial difference in the impact of reservoir levels on the landslide groundwater level. The data of two monitoring locations were selected for establishing the prediction model of groundwater. Combined with the qualitative and quantitative analysis, the influencing factors were selected, respectively, to establish the hybrid Genetic Algorithm-Support Vector Machine (GA-SVM) prediction model. The single-factor GA-SVM without considering influencing factors and the backpropagation neural network (BPNN) model were adopted to make comparisons. The results showed that the multi-factor GA-SVM performed the best, followed by multi-factor BPNN and single-factor GA-SVM. We found that the prediction accuracy can be improved by considering the influencing factor. The proposed GA-SVM model combines the advantages of each algorithm; it can effectively construct the response relationship between groundwater level fluctuations and influencing factors. Above all, the multi-factor GA-SVM is an effective method for the prediction of landslides groundwater in the TGRA.

## 1. Introduction

The groundwater, with storage and migration behaviors, is one of the main natural factors affecting landslide stability. It impacts the various stages of landslide development [1,2]. According to the statistics, 30–40% of dam failures are caused by the damage of groundwater seepage [3], and more than 55% of soil landslides are caused by the effect of groundwater in China. The monitoring and prediction of the landslide groundwater level is a crucial part of landslide early warning systems [4].

The groundwater level in a landslide is mainly affected by external input factors (rainfall, reservoir level, irrigation, etc.) and the permeability of the sliding body (looseness of the soil, development of earth cracks, etc.). For example, for landslides with loose soil and well-developed cracks, the groundwater level clearly rises after rainfall infiltration, which becomes an unfavorable factor for landslide stability [5]. There are mainly three aspects of groundwater that affect the stability of landslides [6,7], including physical and chemical functions of rock and soil, the mechanical function of pore water pressure, and hydrodynamic pressure conditions of landslides. Trigo et al. proposed that landslides of variable movement types are triggered by groundwater level rising and shear strength reduction [8]. Ray and Jacobs and Tommasi et al. also considered that most of the slope failures are caused by soil moisture or groundwater because they increase pore water pressure and decrease shear strength [9,10,11]. It can be seen that the effect of groundwater on landslides is reflected by the change of landslide stability, which is caused by the change of the water level [12]. Hence, real-time process control of groundwater levels can provide an effective judgment on the stability of landslides. 

For the landslides in the Three Gorges Reservoir area (TGRA) in China, under the conditions of water-impoundment of the reservoir, the slopes are recharged directly by groundwater or surface water chronically with strong alternating and cycling actions [13]. The dynamic variation process of the landslide groundwater level is a complex power system. It is controlled by the hydrogeological conditions and the combined action of internal and external factors, such as precipitation and the fluctuation of reservoir water level [14]. However, it is difficult to comprehensively analyze the overall stability of landslides through an accurate analytical method of the measured pore water pressure [15,16]. In general, the change of groundwater level is composed of the deterministic and random components, and the components are influenced by both deterministic and non-deterministic factors. The action rules of these factors will be reflected in the measured data as long as the observation period of the groundwater level is long enough. It is of great significance for landslide forecasting to study the variation characteristics of groundwater under variable conditions of rainfall and reservoir water level, and make a real-time prediction of groundwater level in a potential susceptible landslide. 

In recent years, numerical simulation techniques have been utilized to study the groundwater level change with external factors, including rainfall [17], reservoir impoundment [18], artificial infiltration, drainage, and so on. However, the simulation technique is always complicated, and it requires accurate soil parameters, which are difficult to obtain. Recently, with the development of machine learning (ML) technique, numerous ML models, including hybrid, ensemble, deep learning, etc., have been widely utilized in geology and environment studies [19,20,21]. Choubin et al. applied simulated annealing feature selection to identify key features, and five ML models were used to predict the earth’s fissure hazard [22]. Shamshirband et al. proposed the ensemble models with uncertainty analysis for multi-day ahead forecasting of chlorophyll-a concentration in coastal waters [23]. Lian et al. established a landslide displacement model based on a modified ensemble empirical mode decomposition and extreme learning machine (ELM) [20]. Nguyen et al. presented three novel hybrid ML models for landslide susceptibility modeling, all of which achieved good performance in the area of Vietnam [21]. The advanced ML models have been applied in land subsidence hazard prediction, landslide susceptibility mapping, landslide failure time prediction, and the other fields [22,24,25,26]. However, far too little attention has been paid to the application of ML for the prediction of landslide groundwater level fluctuation. Support Vector Machine (SVM) model was established based on statistical learning theory, which replaced the empirical risk minimization principle of traditional ML methods with the structural risk minimization principle [27]. The SVM model shows a perfect generalization ability to overcome the deficiencies of the traditional artificial neural networks [28,29]. The developed studies present that SVM has achieved excellent performance in both accuracy and stability [25,30,31]. 

A hybrid model can integrate the advantages of each model. Nowadays, using hybrid models has become popular in modeling and estimating studies. These models combine more than one classifier or use one for estimating and the other for parameter optimization. Based on the previous literature, the hybrid model performs better than the single model [32]. SVM advances a lot in dealing with the problem of nonlinear regression prediction. However, its generalization performance is sensitive to the selection of the parameters [33,34,35,36]. The difficulties in capturing the critical modeling variables are noted as the major drawbacks to SVM [22]. Hence, it is urgent to apply optimization algorithms to search for the optimal parameters of SVM. The Genetic Algorithms (GA) is an optimization algorithm; it simulates the mechanism of genetic variation and the theory of biological evolution. GA has the advantages of parallelism and global optimization. It has achieved excellent optimization results in plenty of studies [37,38,39]. In the current study, GA was utilized to select and optimize the parameters of SVM.

Three sensors were installed in Tangjiao landslide to monitor the fluctuation of the groundwater level. Based on the in-suit monitoring data, this study analyzed the variation characteristics of the groundwater level and its relationship with reservoir level and rainfall. Two monitoring positions were selected for establishing a prediction model. The proposed multi-factor GA-SVM, backpropagation neural network (BPNN) model, and the single-factor GA-SVM without considering the factors were utilized to predict the groundwater level of Tangjiao landslide. A comprehensive comparison and assessment of these models will be presented in this study.

## 2. The Forecast Model of Groundwater Level

### 2.1. Proposed Prediction Model 

In the reservoir area, the change of groundwater level in a landslide is associated with geological conditions of the landslide itself, the scheduling of reservoir level, and the rainfall. According to the data of reservoir scheduling, rainfall, and the monitoring data of groundwater level in the Tangjiao landslide, the time-series expression is established as follows:

(1) $$g_{i}=f(x_{i},x_{i+1},\ldots ,x_{i+n},y_{i},y_{i+1},\ldots ,y_{i+n})(i=1,2,\ldots ,n)$$

where *f* is the expression of the SVM, $g_{i}$ is groundwater level, $x_{i}$ is reservoir level, and $y_{i}$ is rainfall. Reservoir level and rainfall are factors independent of each other.

### 2.2. Introduction of the GA-SVM Model

The SVM model is a nonlinear regression forecast method proposed by Vapnik et al. (1995). The input variables are mapped to a high-dimensional linear feature space by the nonlinear transformation. The optimal decision function is constructed. Then the dot product operation of high-dimensional feature space is replaced by the kernel function of the original space. The optimal global solution is obtained by learning and training of finite samples [40]. The regression function is as follows:

(2) f(x)=<W⋅Φ(x)>+b

The estimation function is transformed into a function minimization problem by the insensitive loss function *ε*:

(3) $$R_{\min}=\frac{1}{2}||W|{|}_{}^{2}+C\sum_{i=1}^{m}{\left(\xi_{i}+\xi_{i}{}^{*}\right)}^{}$$

The constraints are as follows:

(4) $$\left\{ \begin{array}{l} W_{}^{T}\phi (x_{i})+b_{i}-y_{i}\leq \varepsilon +\xi_{i};\\ y_{i}-W_{}^{T}\phi (x_{i})-b_{i}\leq \varepsilon +\xi_{i}^{*};\\ \xi_{i},\xi_{i}^{*}\geq 0,i=1,\cdots ,l. \end{array}\right.$$

where, C is the penalty factor, $\xi_{i}$ and $\xi_{i}^{*}$ are relaxation factors, b is the offset value. Finally, by introducing Lagrange multipliers and the application of Wolf duality theory, it is translated into an equivalent dual problem as follows:

(5) $$\min\frac{1}{2}{\left(\alpha -\alpha_{}^{*}\right)}_{}^{T}Q(\alpha -\alpha_{}^{*})+\varepsilon \sum_{i=1}^{l}(\alpha_{i}^{}+\alpha_{i}^{*})+\sum_{i=1}^{l}y_{i}(\alpha_{i}^{}-\alpha_{i}^{*})$$

The constraints are as follows:

(6) $$\left\{ \begin{array}{l} \sum_{i=1}^{l}(\alpha_{i}+\alpha_{i}^{*})=0;\\ 0\leq \alpha_{i},\alpha_{i}^{*}\leq C,i=1,2\cdots l. \end{array}\right.$$

where, $Q_{ij}=K(x_{i},x_{j})=f{\left(x_{i}\right)}^{T}f(x_{j})$.

By quadratic programming, the regression forecast model is obtained as follows:

(7) $$f(x,\alpha_{i}^{*},\alpha_{i}^{})=\sum_{i=1}^{l}\left(\alpha_{i}^{*}-\alpha_{i}^{}\right)K(x_{i},x)+b$$

where, $K(x_{i},x)$ is the kernel function of the SVM. At present, there are four commonly used kernel functions—linear kernel function, polynomial kernel function, radial basis kernel function (RBF), and sigmoid function.

The genetic algorithm is an artificial intelligence algorithm. It searches the optimal solution by simulating the process of natural evolution. This algorithm simulates phenomena that occurred in natural selections and genetics, such as reproduction, crossover, and mutation. Starting from an initial population, through the operation of selecting, crossing and mutating, a group of better-adapted individuals is produced. These individuals make the group evolve into a better area in the search space. Then through the constant multiplications and evolutions, the optimal solution can be obtained by converging to the individuals, which can best adapt to the environment.

**Figure 1:**
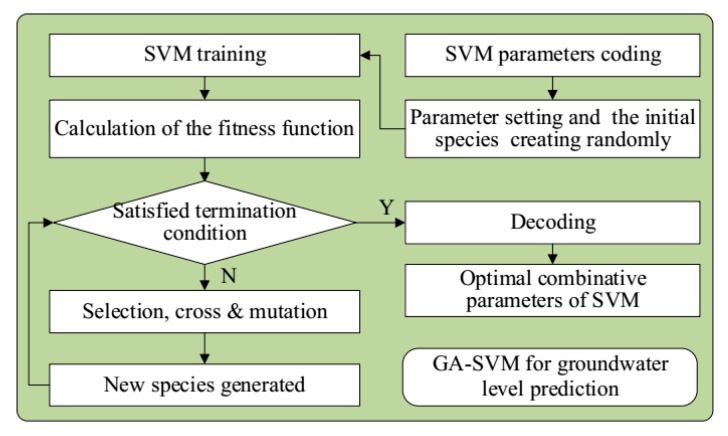
Analysis flowchart of the GA-SVM prediction model.

Considering that the SVM is sensitive to the model parameters, GA was adopted to optimize the parameters, and the GA-SVM coupling model was established. The flowchart is depicted in Figure 1.

### 2.3. Introduction of the BPNN Model 

**Figure 2:**
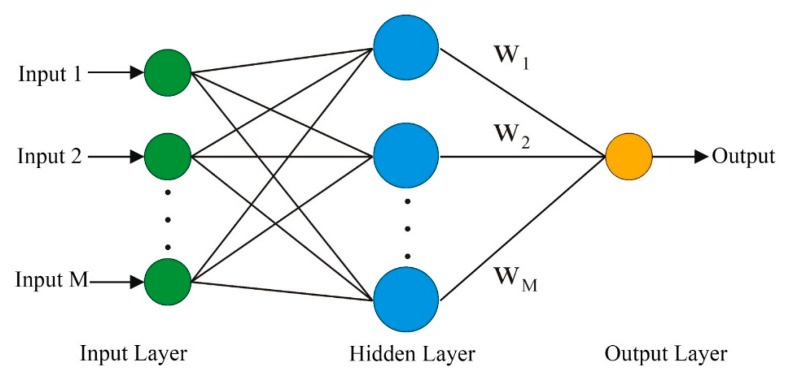
The basic structure of BPNN.

BPNN is a multilayer feedforward neural network. It has the characteristics of error reverse transmission and signal forward transmission. BPNN is learned by example, which consists of some input examples and the known correct output for each case. In the first step of the BPNN process [41], the learning samples are added to the input network. Then, the back-propagation algorithm is used to train the weights and deviations in the network. It more closely matches the output vectors and expectation vectors. If there is a big error between the output result and the expected result, the error propagates backward, adjusts the network threshold and weight, and is repeated to eventually make the predicted output result approach the expected output result. The topological structure of BPNN is shown in Figure 2. Previous research has shown that the basic architecture of a three-layered neural network consists of an input layer, a hidden layer, and an output layer. It can accurately fit nonlinear mapping relationships [42]. Additionally, the trial-and-error approach is used to choose an appropriate number of hidden neurons in the BPNN model.

### 2.4. Evaluation Model of the Prediction Accuracy

Root mean square error (*RMSE*, Equation (8)) and the mean absolute percentage error (*MAPE*, Equation (9)) are commonly used indexes for accuracy testing of prediction models. The smaller the values of *RMSE* and *MAPE* are, the better the prediction result is. However, the values of *RMSE* and *MAPE* are associated with the value of the motoring data. The correlation coefficient (Equation (10)) is not related to the monitoring data; it can reflect the correlation between the variables. This paper has adopted the correlation coefficient for the forecast accuracy analysis [43].

(8) $$RMSE=\sqrt{\frac{1}{N}\sum_{i=1}^{N}{\left({\hat{x}}_{i}-x_{i}\right)}_{}^{2}}$$

(9) $$MAPE=\frac{1}{N}\sum_{i=1}^{N}\left|\frac{x_{i}-{\hat{x}}_{i}}{x_{i}}\right|$$

(10) $$R=\frac{\sum_{i=1}^{N}\left(x_{i}-\bar{x})({\hat{x}}_{i}-\bar{\hat{x}}\right)}{\sqrt{\sum_{i=1}^{N}{\left(x_{i}-\bar{x}\right)}_{}^{2}}\sqrt{\sum_{i=1}^{N}{\left({\hat{x}}_{i}-\bar{\hat{x}}\right)}_{}^{2}}}$$

where, $x_{i}$ is the measured value; ${\hat{x}}_{i}$ is the predicted value; N is the number of predicted values; $\bar{x}$ is the mean of the measured values; $\bar{\hat{x}}$ is the mean of predicted values.

## 3. Sensors Installation and Data Acquisition

### 3.1. Geologic Conditions of the Monitored Landslide—The Tangjiao Landslide

**Figure 3:**
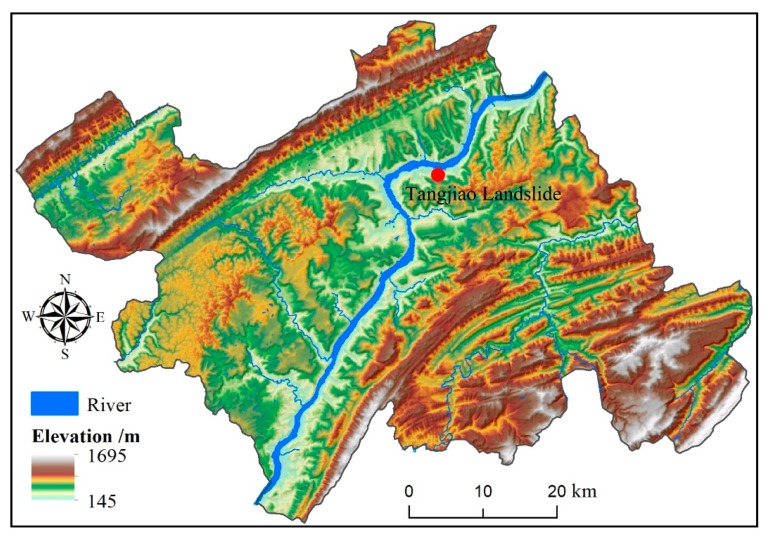
Location map of the Tangjiao landslide in the Wanzhou district.

The Tangjiao landslide is located in the Wanzhou district of Chongqing City, the east edge of the Sichuan basin and the south side of the Yangtze River (Figure 3). Once the landslide fairs, it will endanger the lives and property of 709 people in the landslide area. It will also threaten the safety of the road and shipping of the Yangtze River.

**Figure 4:**
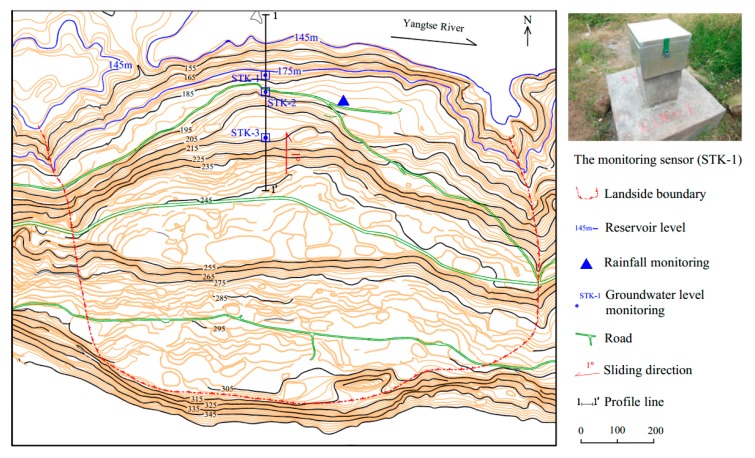
Topographical map of the Tangjiao landslide, with monitoring points.

The Tangjiao landslide location belongs to the bank accumulation landforms of the Yangtze River valley. In the front of the landslide, the terraces and flood plains develop well with gentle step surfaces. They bulge out like the tongue and show arc shapes in contours. The slope inclines with radial shape. The middle and rear part of the landslide is gentler than the front. The back edge of the landslide is concave southwards with cirque shape. the slope gradient is 25°–45° with many bedrocks exposed. The landslide is an oversized soil deformation. It is fan-shaped on the whole platform, with the main sliding direction of 359°. It has an estimated volume of 2672.4 × 10^4^ m^3^, with an average thickness of 20 m. The entire landslide covers an area of 133.62 × 10^4^ m^2^, with a maximum longitudinal dimension of 1020 m and an average width of 1310 m (Figure 4). 

**Figure 5:**
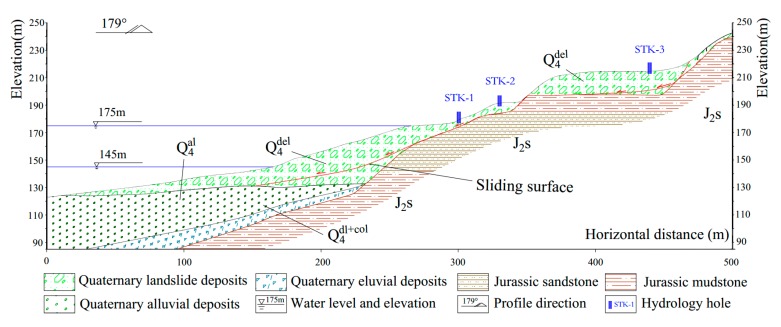
Schematic Ⅰ-Ⅰ’ geological cross-section of Tangjiao landslide.

**Figure 6:**
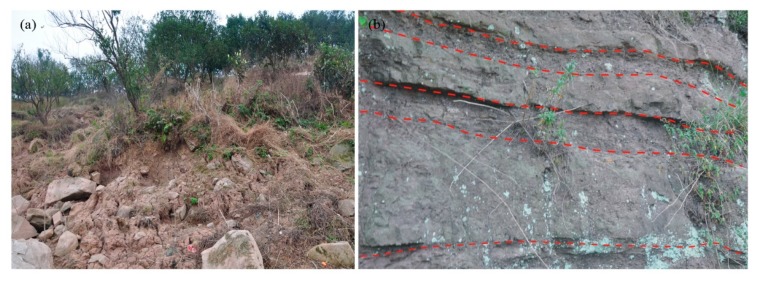
(**a**) Deposits with rubble and block stones; (**b**) Bedrock interbedded with sandstones and mudstones.

The landslide is located in the core area of Wanxian syncline. Its axis trend is NE30°–60°. The attitude of the core rock layer is 153°∠4° with two asymmetrical wings, the steep northwest one (40°–80°) and the gentle southeast one (15°–40°). The landslide is locally covered by thick gravelly soil accumulated from quaternary collapse-slide and eluvial-colluvial deposits. The underlying bedrock is interbedded with sandstone and mudstone of the Shaximiao Formation of Middle Jurassic, the strata attitude of which is 170°∠5° (Figure 5 and Figure 6).

### 3.2. In-Suit Monitoring and Data Acquisition

**Figure 7:**
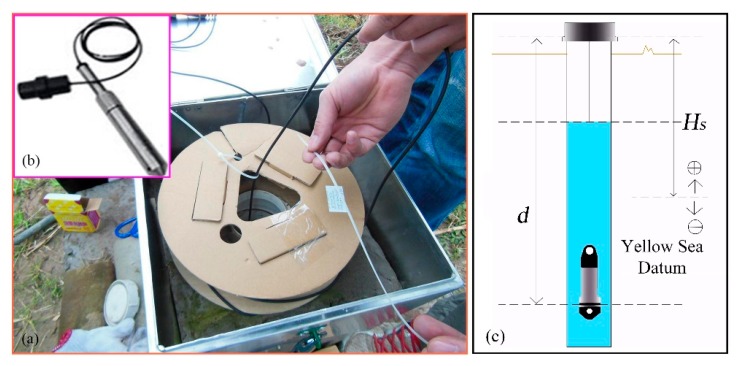
The principle and installation of groundwater level monitoring device (**a**) Sensor installation (**b**) a sensor for monitoring and (**c**) the principle of monitoring.

The principle and in-suit installation of groundwater level monitoring device are shown in Figure 7.

The formula of the groundwater level is shown below:

(11) $$H=h/100-h_{s}+H_{s}-d$$

where, *H* is the groundwater level; *h* is the device records(cm); *h_s_* is the height of water column at standard atmospheric pressure, *h_s_* = 10.336 m; *H_s_* the elevation of the hydrologic hole(m); *d* is the distance between monitoring probe and hydrologic hole, in this paper, *d* = 5 m.

**Figure 8:**
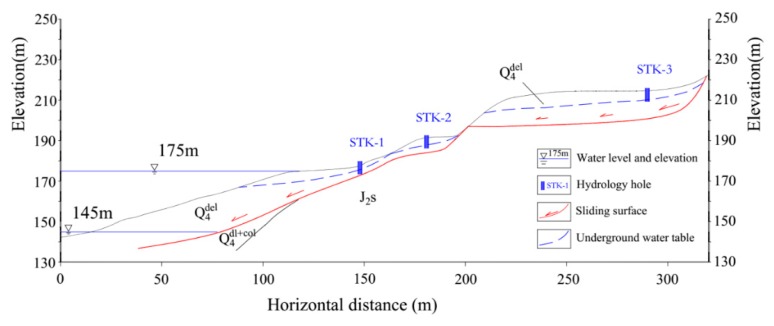
Generalized schematic section of groundwater in the toe of the landslide.

Three groundwater monitoring points (STK) were installed on the Tangjiao landslide. They were respectively labelled as STK-1, STK-2, and STK-3, and their positions are shown on Figure 4, Figure 5 and Figure 8. 

**Figure 9:**
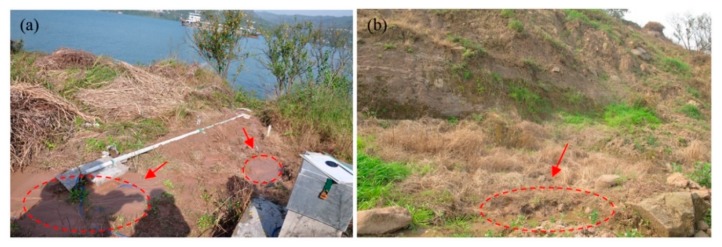
(**a**) Water exuded on the ground near STK-1; (**b**) Water exuded on the ground near STK-2.

**Figure 10:**
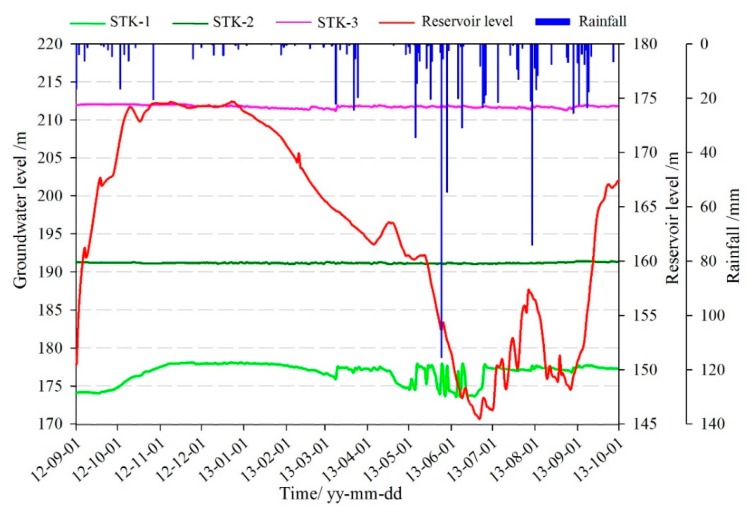
Monitoring curves of groundwater level of the Tangjiao landslide.

The three monitoring points of groundwater level were all installed on the terrace III. The groundwater level of STK-1 varied obviously along with the fluctuation of the reservoir level, while the STK-2 and the STK-3 almost remained unchanged (Figure 8). STK-1 is located on the toe of the front part with an elevation of 179 m, and it is close to the river; therefore, the groundwater can be directly recharged or discharged by the reservoir water. The elevations of the STK-2 and STK-3 are 192 m and 213 m, respectively. The STK-3 is located on the second platform of the junction of terrace III and IV. The maximum height of the reservoir level is about 175 m, which shows less impact on the groundwater of the two positions. From field investigations, it was found that during the high reservoir level season, groundwater is often exposed to small scarps (Figure 9a); and similar results were found for the STK-2 in rainy seasons (Figure 9b). Consequently, it is reasonable to infer that the groundwater in the toe of the Tangjiao landslide consists of different hydrologic systems. The groundwater of STK-1 and STK-2 are influenced both by the reservoir level and rainfall. The reservoir level is the most obvious factor for STK-1, while STK-3 is mainly influenced by rainfall. The groundwater level of STK-1 and STK-3 were used to establish the prediction model in this paper (Figure 10).

## 4. The Groundwater Level Prediction of STK-1

### 4.1. Monitoring Data of Groundwater Level

**Figure 11:**
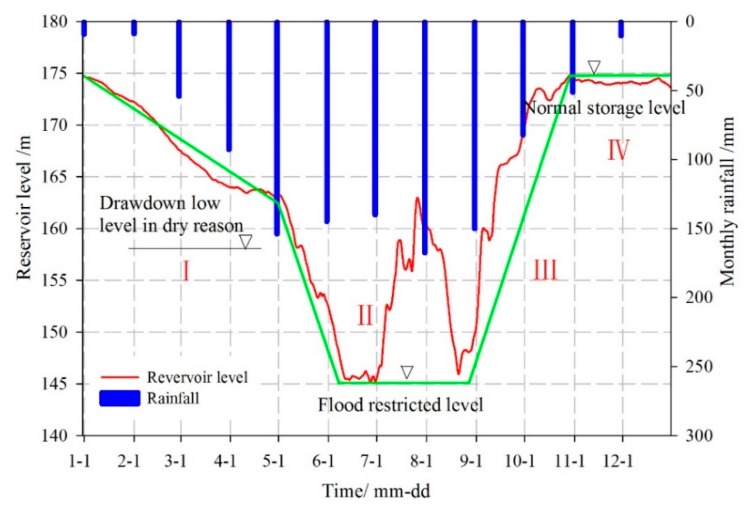
Water level regulation of the Three Gorges Reservoir in 2012 [36].

The water-impoundment of the Three Gorges Reservoir has reached full capacity since 2007. The fluctuation of the reservoir level ranges from 145 m to 177 m with a variation of 30 m. The scheduling process is divided into four stages (Figure 11). It makes the hydrogeological conditions of the reservoir area in Wanzhou represent different characteristics in different periods during the year [44].

**Figure 12:**
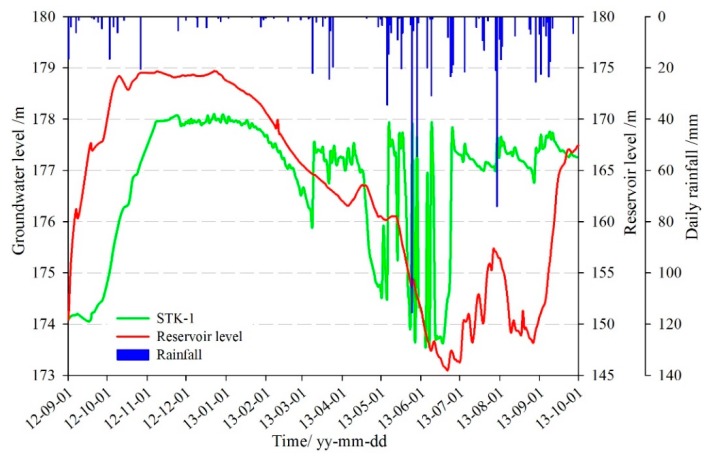
Monitoring curves of groundwater level in STK-1.

The groundwater level with time was analyzed and summarized below (Figure 12):

(1) Between September 2012 and May 2013, the fluctuation of the reservoir level maintained a high degree of consistency with the groundwater level of hydrological hole STK-1. From May to July 2013, the reservoir level was continuously decreasing from 160 m to 145 m, while the groundwater level clearly fluctuated under the influence of rainfall. The reason is that the front part of the landslide is covered by loose deposits, the high permeability of the soil, and the penetrative pressure induced by short-time intensive rainfall make the groundwater recharge quickly by the rainfall. So, the continuous rise of the groundwater level was affected by the rainfall more than the reservoir level during this period.
(2) On 25 May 2013, the rainfall reached 115 mm/d, and the groundwater level rose from 173.5 m to 177.5 m which increased 4 m within 24 h. Meanwhile, the reservoir level fell at a speed of 1.1 m/d. Three days after the rain stopped, the groundwater level dropped to 174 m again, and then rose rapidly when the rainfall reached 54.6 mm/d on 29 May.
(3) Between September and November 2013, the reservoir level increased into the normal storage level of 175 m, but the groundwater level showed a slight downward trend.

The characteristics of the groundwater level in STK-1 indicated that the groundwater maintained close hydraulic connection and significant response relationship with the reservoir level and rainfall. They were the main factors of the changes in the groundwater level. Due to the geological conditions of the landslide, there was a hysteresis effect when the groundwater level was changing with the variation of rainfall and reservoir level, but the trends were in agreement substantially. When the reservoir level rose, and the rainfall increased, the groundwater would be recharged to a corresponding uplift. On the contrary, when the reservoir level dropped, and the rainfall decreased, the groundwater level would decline.

### 4.2. Determination of Influencing Factors

The variable speed of the groundwater level is related to the fluctuation speed of reservoir level and the permeability of the soil in front of the landslide. The greater the speed of groundwater level declines, the faster the decrease of the seepage line in a landslide will be. When the reservoir level drops at a certain speed and the rains last in a certain duration, the position of the seepage line would depend on the rainfall intensity. With the changes in the external conditions and the soil permeability, the response of the groundwater level shows hysteresis effects in various degrees.

**Figure 13:**
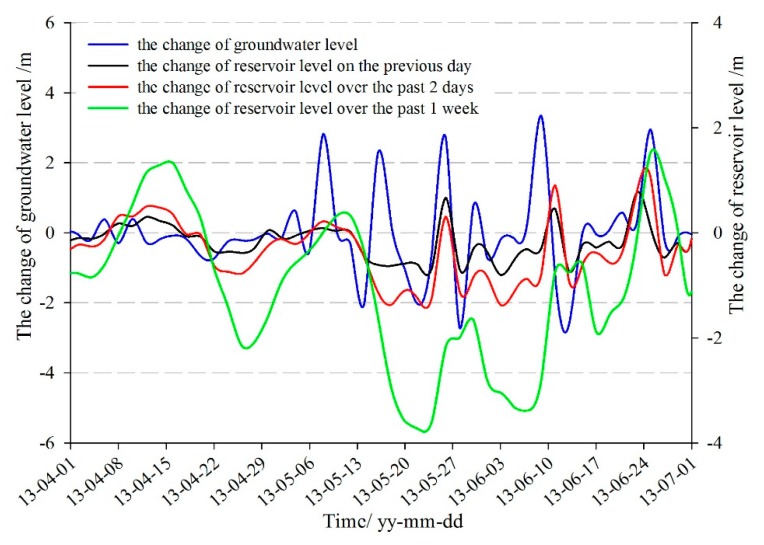
The relation between the groundwater level and the reservoir level.

The hydrology hole STK-1 is located on the front part of the Tangjiao landslide at a lower altitude. The soil is loose with large permeability coefficient, so the lag-time, the groundwater response to the reservoir water, is shorter than other parts of the landslide. The monitoring data in Figure 12 and Figure 13 showed that the shape of the change rate curves of the groundwater level in the Tangjiao landslide was similar to that of the change of the reservoir level on the previous day, the change of the reservoir level over the past two days, and the change of the reservoir level over the past one week. Hence, the reservoir level on the current day, the change of the reservoir level on the previous day, the change of the reservoir level over the past two days, and the change of the reservoir level over the past one week were selected as the reservoir level factors of groundwater level.

**Figure 14:**
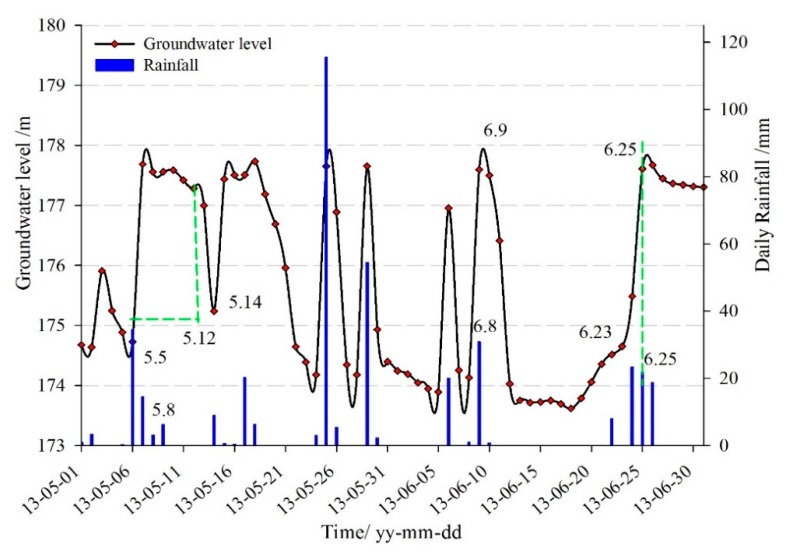
The relationship between rainfall and groundwater level from May to July.

Figure 14 showed that it was at a lower level of the reservoir from May to July, and the rainfall period as well. So, besides the fluctuation influence of the reservoir level, the groundwater level was also influenced by the rainfall. The groundwater increased within 1–2 days when heavy rainfall occurred, which was seen from 8–9 June. The relationship between the groundwater level and the rainfall during 5–8 May and 23–25 June showed that, when the continuous heavy rainfall occurred, the groundwater rose rapidly and was maintained at a high level, about one week after the rainfall stopped. Then because of the geotechnical characteristics of the landslide, surface evaporation, and flow discharge, the groundwater returned to the initial state rapidly within a short period. The continuous heavy rainfall was the main reason for the rise of the groundwater level.

The degree of grey correlation is applied to make a dynamic analysis on the degree of similarity or dissimilarity of the development trend of multi-variable nonlinear time series. It is a quantitative index for measuring the correlation. As the relationship between the groundwater level and its factors was dynamically changing with time, the calculation of the absolute difference and the degree of grey correlation were performed through the normalized processing. The values of grey correlation are shown in Table 1.

The results showed that the degree of grey correlation between the influencing factors and the groundwater level were all greater than 0.800. It indicated a great consistency of the changing tendency. The influence of the reservoir level was stronger, which was in a valid agreement with the monitoring data. 

This paper selected the reservoir level on current day, the change of the reservoir level on the previous day, the change of the reservoir level over the past two days, the change of the reservoir level over the past one week, the rainfall on current day, the cumulative rainfall on the previous day, the cumulative rainfall over the past two days and the cumulative rainfall over the past one week as the influencing factors to establish the response model between the factors and the groundwater level and to predict the groundwater level in the STK-1.

### 4.3. The Result Analysis of the Prediction Model

The monitoring data from 15 February to 7 June 2013 were selected as the training sample of the time-series for the GA-SVM prediction model. The data from 9 to 27 June 2013 were selected as the test sample, which was within the period when the groundwater level fluctuated the most.

In order to eliminate the influence of the dimension, a normalization processing was applied to all the data, and the normalization formula is shown as follows:

(12) $$x=\frac{X-X_{min}}{X_{max}-X_{min}}$$

where, *x* are the normalized values, *X* are the original values, *X_max_* is the maximum value of a sequence, and *X_min_* is the minimum value of a sequence.

**Figure 15:**
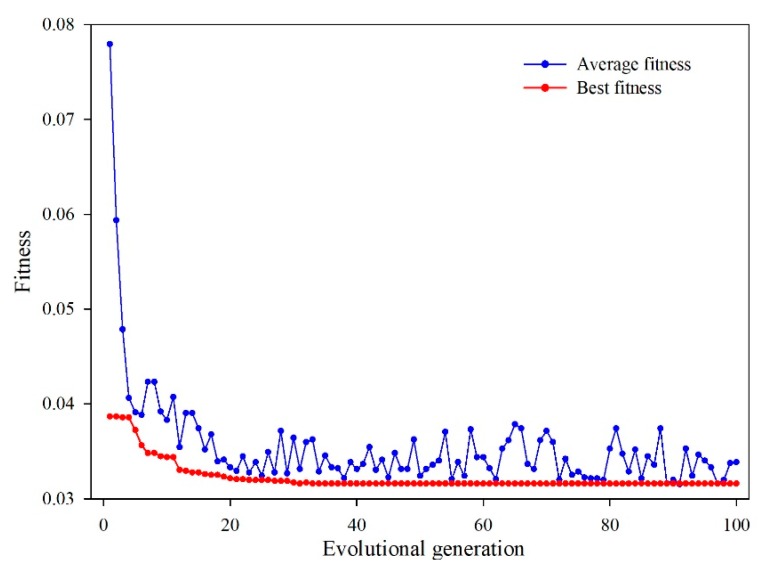
The parameters searching process of GA.

The population of the GA was set as pop = 30 and the maximum iterative steps number was 100. The optimal parameters of the penalty factor C and the radial basis kernel function (RBF) γ were searched by the GA (Figure 15), which was C=6.9709 and γ=0.58537, respectively. Using the optimal parameters, the multi-factor SVM model was established to find the response relationship between the groundwater level and the influencing factors. The model was trained by the training sample, then the trained model was used to make a prediction of the groundwater level.

**Figure 16:**
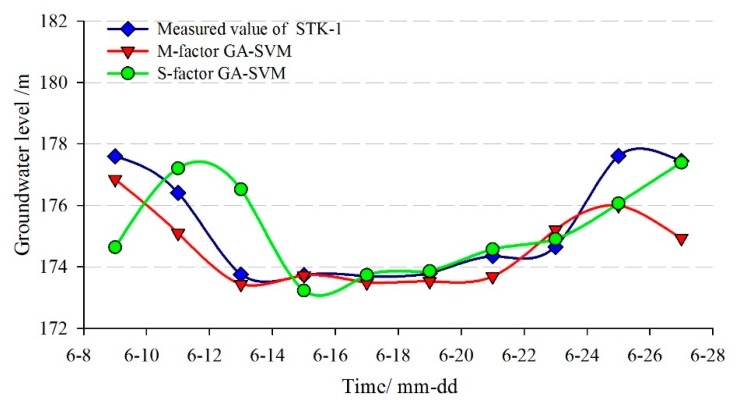
The predicted result of groundwater level in STK-1 by the GA-SVM model.

**Figure 17:**
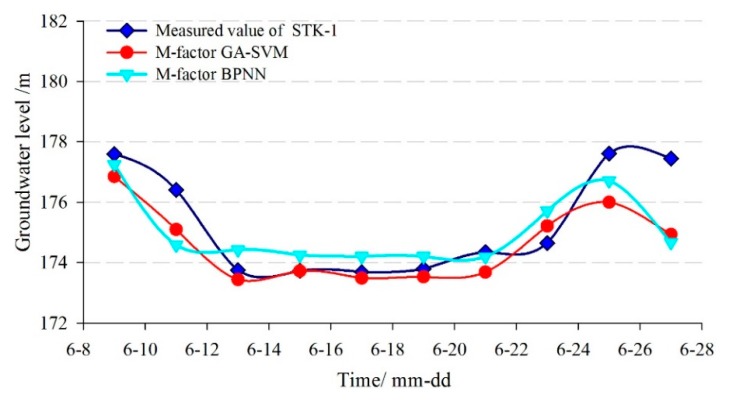
The predicted result of groundwater level in STK-1 by the BPNN model.

In addition, the single-factor SVM prediction model without considering the factors and the BPNN model were adopted to make a comparative analysis with the proposed multi-factor model. The comparison curves between the predicted and measured groundwater levels are shown in Figure 16 and Figure 17. The evaluation results of the prediction accuracy are shown in Table 2.

Figure 16 and Figure 17 showed that the predicted results of the three models were all consistent with the trend of measured values, but the prediction error at the turning points was relatively larger using the single-factor model. 

Table 2 showed that the *RMSE* and the *MAPE* of the multi-factor GA-SVM model were 1.104 m and 0.465%, while the values of the multi-factor BPNN model were 1.195 m and 0.522%, respectively. The correlation coefficient (*R*) of the former was 0.881, which was greater than the latter (0.718). So, the multi-factor GA-SVM model outperforms the multi-factor BPNN model. The *RMSE*, *MAPE*, and *R* of the single-factor GA-SVM model were 1.409 m and 0.525%, 0.591, respectively. The prediction accuracies of the multi-factor models were much greater than the single-factor model, so the consideration of the influencing factors in the multi-factor model can make the results superior to that of the single-factor model. The main reasons are, on the one hand, the groundwater level of the landslide is under influencing factors such as reservoir level and rainfall. On the other hand, because of the complex geological conditions of the Tangjiao landslide, there is a complicated nonlinear relationship between the groundwater level and its influencing factors. In conclusion, the multi-factor prediction model considering the factors that showed a higher prediction accuracy. 

## 5. The Groundwater Level Prediction of STK-3

### 5.1. Monitoring Data of Groundwater Level

**Figure 18:**
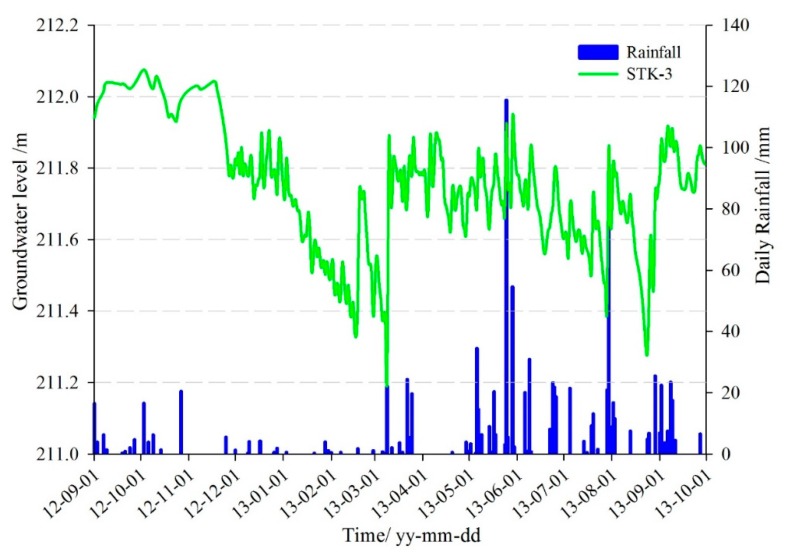
Monitoring curves of groundwater level in STK-3.

As can be seen from the monitoring curves (Figure 18), the rainfall reached 115 mm/d on 25 May 2013, the groundwater level rose from 211.6 m to 211.9 m, which increased 0.3 m within 24 h. Three days after the rain stopped, the groundwater dropped to 211.7 m again. Then it rose rapidly when the rainfall reached 54.6 mm/d on 29 May. Although the groundwater level of STK-3 fluctuating within a narrow range, it was because of high sensitivity to rainfall.

### 5.2. Determination of Influencing Factors

The changes of groundwater level of STK-3 was mainly caused by rainfall. It was hardly influenced by the reservoir level. So, the rainfall was selected as the factor for the groundwater level prediction of STK-3. The degree of grey correlation between the groundwater level and the factors are shown in Table 3.

The results show that the groundwater level kept a great consistency of changing tendency with the influencing factors, which was in a valid agreement with the monitoring data. This paper selected the rainfall on the current day, the cumulative rainfall on the previous day, the cumulative rainfall over the past two days, and the cumulative rainfall over the past one week as the influencing factors to predict the groundwater level of STK-3.

### 5.3. The Result Analysis of the Prediction Model

**Figure 19:**
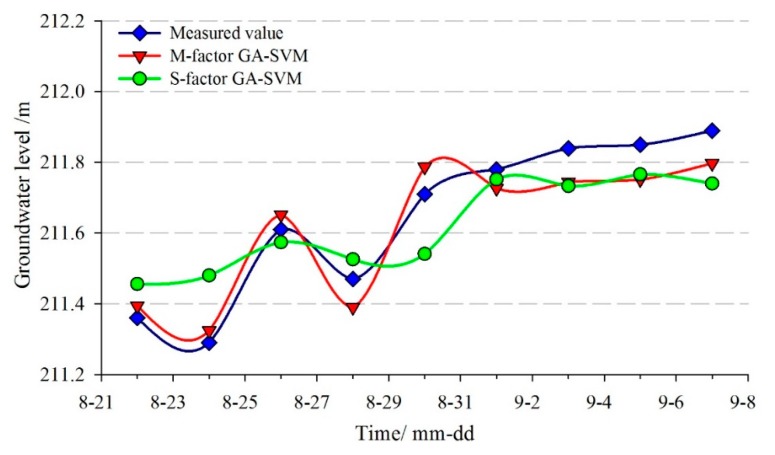
The predicted result of groundwater level in STK-3 by GA-SVM model.

**Figure 20:**
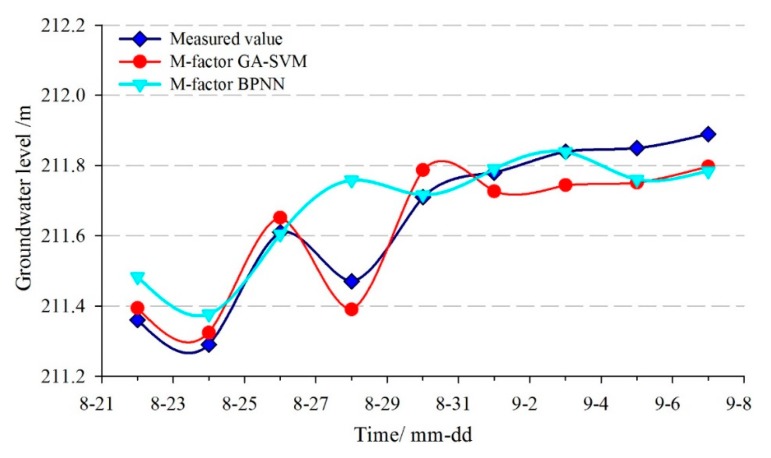
The predicted result of groundwater level in STK-3 by BPNN model.

The monitoring data from 20 January to 20 August 2013, were selected as the training sample of the time-series prediction model for STK-3. The data from 22 August to 7 September 2013, were selected as the test sample. The population of the Genetic Algorithm was set as pop=35 and the maximum iterative steps number was 35. The optimal parameters of the penalty factor *C* and the radial basis kernel function γ were searched by Genetic Algorithm, which was C=4.8183 and γ=30.0069, respectively. The comparison curves between the predicted and measured groundwater levels are shown in Figure 19 and Figure 20, and the evaluation results of the prediction accuracy are shown in Table 4.

Figure 19 and Figure 20 showed that the goodness-of-fit of the multi-factor GA-SVM model was greater than the single-factor GA-SVM model and the multi-factor BPNN model, especially when the strong rainfall occurred and the groundwater level was fluctuating (24–31 August). The prediction results of the multi-factor GA-SVM model were closer to the measured values. Table 4 shows that the *RMSE* and the *MAPE* of the multi-factor GA-SVM model were 0.072 m and 0.032%, while the values of the BPNN model were 0.117 m and 0.0376%, respectively. The correlation coefficient of the former was 0.953; it was greater than the latter (0.860). Compared with the multi-factor models, the *RMSE*, *MAPE*, and *R* of the single-factor GA-SVM model were 0.116 and 0.048%, 0.914, respectively. The prediction results of the multi-factor model were superior to that of the single-factor model. These suggest that based on deep analysis of the geological conditions of landslide and selecting the appropriate factors, an accurate prediction of the groundwater level can be achieved.

## 6. Discussion

### 6.1. Performance Comparison of GA-SVM and BPNN

**Figure 21:**
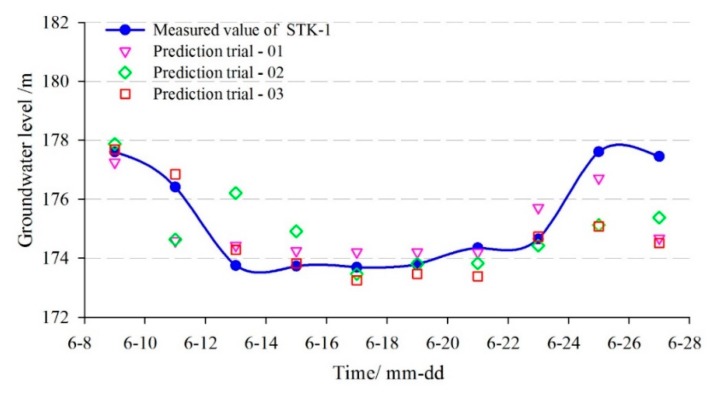
The prediction accuracy comparisons between different trials of BPNN(STK-1).

**Figure 22:**
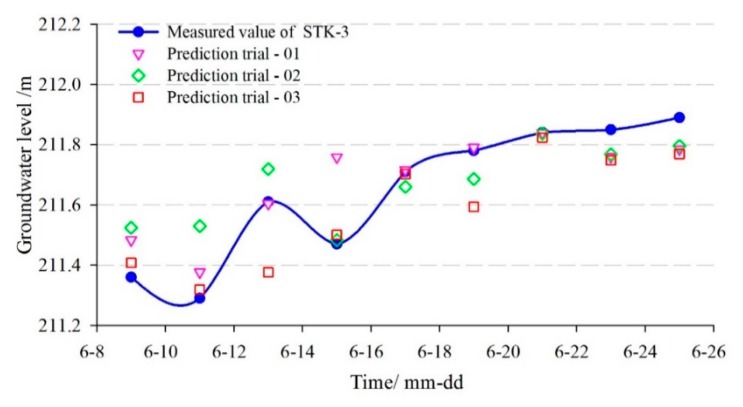
The prediction accuracy comparisons between different trials of BPNN(STK-3).

In order to compare the prediction stability of the GA-SVM and BPNN, three sets of prediction experiments were obtained with the same inputs and model parameters (Figure 21 and Figure 22). The REMS of prediction results are shown in Table 5. Although the inputs and parameter of the BPNN are the same, three sets of predicted results are different, while the prediction results of GA-SVM is constant. Overall, the proposed multi-factor GA-SVM method can achieve accurate and stable predictions in the groundwater level of landslides.

### 6.2. Future Developments of ML Methods

The groundwater level is a key parameter in the assessment of landslide stability. Accurate monitoring and prediction of groundwater level are essential for landslide early warning. Machine learning is a very effective prediction technology. It has been applied to landslide prediction and has achieved excellent results, such as landslide susceptibility mapping. In order to reduce the dependence on tedious numerical simulations, how to make full use of ML technology for landslide groundwater level prediction needs to be studied further.

There have been few reports on the application of ML techniques to groundwater level prediction up to now. We compared the performance of ML models in the application, mainly the accuracy and stability of prediction. In this study, through an optimum parameter searching by the GA optimization algorithm, a hybrid model of the coupled SVM and GA algorithm was established for prediction. Although GA-SVM performed well, in future studies, it is necessary to research the prediction performance of more advanced ML algorithms, such as ELM, Adaptive Network-based Fuzzy Inference System, optimization algorithms (Particle Swarm Optimization, Artificial Bee Colony, Grey Wolf Optimizer, etc.), and ensemble learning (Bagging, Boosting, Stacking, etc.), Etc.. In order to achieve an accurate and stable prediction of landslide groundwater level, its urgent to study the combination of various algorithms and integrate their advantages.

### 6.3. Geological Factors of Groundwater Level 

The groundwater level is not only affected by external inputs (rainfall, reservoir level, irrigation, etc.), but also closely related to the appearance condition of the slope, including the landslide structure and the macroscopic deformation characteristics (earth cracks, etc.). During the development process of a landslide, earth cracks are important companion products [45]. The surface cracks destroy the integrity of the soil, and significantly change the infiltration path and the distribution of the seepage field as well. They provide a preferential flow path for the rapid migration of water to deep soil. The study found that a slope would maintain a high saturation under long-term continuous rainfall, and the top-down development of the wetting front would eventually lead to direct precipitation of groundwater [46]. For landslides with loose mass and well-developed cracks, the groundwater will fill the cracks rapidly in a short-term concentrated rainfall. The anisotropy of crack parameters has different influence rules and degrees on groundwater level. The larger the permeability coefficient corresponding to the parameters, including the location, width, depth, density, length, fracture size, and suction characteristics of earth cracks, the faster the groundwater responses. Hence, how to predict the groundwater level by combining the characteristics of the influencing factors, the sliding body and the macroscopic deformation of a landslide needs further study.

## 7. Conclusions

The monitoring and prediction of the groundwater level are significant for landslide early warning. In this study, the Tangjiao landslide in the TGRA was taken as an example. Three groundwater level monitoring sensors were installed in different locations of the landslide. Based on the monitoring data, the response relationship between landslide groundwater level fluctuations and factors is analyzed, multi-factor GA-SVM, multi-factor BPNN, and single-factor GA-SVM prediction models were established. The main conclusions are as follows: (1) The fluctuation of groundwater level is significantly consistent with rainfall and reservoir level in time, and there is also a lag. There is a spatial difference in the impact of reservoir level on the groundwater level of the reservoir landslide. The closer to the Yangtze River, the stronger the reservoir level effect and the shorter the lag time; (2) On the basis of qualitative and quantitative analysis, the model inputs of STK-1 and STK-3 were established, respectively. Comparing the prediction results of multi-factor GA-SVM and single-factor GA-SVM; it can be found that multi-factor GA-SVM has higher accuracy at both locations. In the establishment of landslide groundwater level prediction model, it can significantly improve the prediction accuracy to consider the response of factors; (3) Comparing the prediction results of the multi-factor GA-SVM and the multi-factor BPNN models, it demonstrated that the prediction accuracy and stability of the GA-SVM model are both superior to the traditional BPNN model. The proposed GA-SVM model integrates the advantages of GA and SVM, it shows excellent modeling efficiency and accuracy. In general, by combining the advantages of different artificial intelligence algorithms, the multi-factor GA-SVM model proposed in this paper can effectively construct the response relationship between groundwater level fluctuations and influencing factors. It is a reliable prediction model, which can be recommended in predicting landslide groundwater levels.

## Figures and Tables

**Table 1:** Calculation results of grey correlation between groundwater level and factors of STK-1.

Influencing Factor	Degree of Grey Correlation
The reservoir level on the current day	0.988
The change of the reservoir level on the previous day	0.827
The change of the reservoir level over the past two days	0.827
The change of the reservoir level over the past one week	0.828
The rainfall on the current day	0.854
The cumulative rainfall on the previous day	0.854
The cumulative rainfall over the past two days	0.859
The cumulative rainfall over the past one week	0.879

**Table 2:** Evaluation results of the prediction accuracy of STK-1.

Prediction Model		Multi-Factor GA-SVM	Single-Factor GA-SVM	Multi-Factor BPNN
Accuracy	*RMSE*/m	1.104	1.409	1.195
	*MAPE*/%	0. 465	0.525	0.522
	*R*	0.881	0.591	0.718

**Table 3:** Calculation results of grey correlation between groundwater level and factors of STK-3.

Influencing Factor	Degree of Grey Correlation
The rainfall on the current day	0.856
the cumulative rainfall on the previous day	0.856
the cumulative rainfall over the past two days	0.860
the cumulative rainfall over the past one week	0.880

**Table 4:** Evaluation results of the prediction accuracy of STK-3.

Prediction Model		Multi-Factor GA-SVM	Single-Factor GA-SVM	Multi-Factor BPNN
Accuracy	*RMSE*/m	0.072	0.116	0.117
	*MAPE*/%	0.032	0.048	0.0376
	*R*	0.953	0.914	0.860

**Table 5:** The prediction accuracy comparisons between different trials of BPNN.

	STK-1					STK-3				
Model	1st	2nd	3rd	Mean	Variance	1st	2nd	3rd	Mean	Variance
BPNN	1.195	1.466	1.295	1.319	0.112	0.118	0.117	0.115	0.117	0.001
GA-SVM	1.104	1.104	1.104	1.104	0.000	0.072	0.072	0.072	0.072	0.000
